# COVID-19-Associated Mucormycosis in a Tertiary Care Hospital in India: A Case Series

**DOI:** 10.7759/cureus.27906

**Published:** 2022-08-11

**Authors:** Shivam Singh, Pragati Basera, Aditya Anand, Ahmad Ozair

**Affiliations:** 1 Internal Medicine, King George's Medical University, Lucknow, IND; 2 Neurosurgery, King George's Medical University, Lucknow, IND

**Keywords:** covid-19 epidemiology, public health, liposomal amphotericin b, covid-19 india, rhinocerebral mucormycosis

## Abstract

Mucormycosis is a disease that usually occurs in immunocompromised patients or those with uncontrolled diabetes mellitus. The second wave of the coronavirus disease 2019 (COVID-19) pandemic in India was accompanied by an unexpected rise in mucormycosis cases, ranging from the most commonly occurring Rhino-orbital-cerebral mucormycosis (ROCM) to rare cases of pulmonary and gastrointestinal mucormycosis. The majority of cases that presented to our hospital were individuals with underlying diabetes mellitus who received steroids for COVID-19 before being diagnosed with mucormycosis.

In this case series, we present five rhino-orbital-cerebral mucormycosis cases that were histopathologically positive and treated at a tertiary-care hospital in India. Magnetic resonance imaging (MRI) of all of the patients demonstrated orbital apex syndrome and diffuse or focal infiltration of the cavernous sinus. Cases were treated with anti-fungal drugs, transcutaneous retrobulbar injection of amphotericin B (TRAM B), along with appropriate surgical excision and debridement of the involved tissue.

The essential elements for successfully managing this fatal infection are control of the predisposing factors, early detection, anti-fungal drugs, and surgical debridement of the involved tissues.

## Introduction

Mucormycosis is a fatal angioinvasive infection by the fungal species of the order Mucorales, which include genera of ubiquitous fungi such as *Mucor *and *Rhizopus* [1].

Mucormycosis is characterised by infarction and necrosis of the host tissues. It is classified based on anatomical sites of involvement, such as rhino-cerebral, pulmonary, gastrointestinal, cutaneous, and disseminated forms. The most common form is rhino-cerebral mucormycosis, which is spread by the inhalation of spores to a host with predisposing factors, which include diabetes mellitus, particularly ketoacidosis, treatment with glucocorticoids, hematologic malignancies, haematopoetic stem-cell or solid organ transplantation, iron overload, and malnutrition. The diagnosis of mucormycosis relies upon the identification of organisms in tissue by histopathology with culture confirmation [1-9].

An unexpected rise of mucormycosis cases accompanied the second wave of the coronavirus disease 2019 (COVID-19) pandemic in India. The majority of cases that presented to our hospital were individuals with underlying diabetes mellitus who received steroids for COVID-19 before being diagnosed with mucormycosis. Rhino-orbital-cerebral mucormycosis was most commonly noted, manifesting as facial pain, facial or orbital swelling, headache, and/or nasal eschar.

Treatment of mucormycosis involves a combination of surgical debridement of involved tissues and anti-fungal therapy [10,11]. Intravenous (IV) amphotericin B (a lipid formulation) is the drug of choice for initial therapy [12,13]. Posaconazole or isavuconazole is used as step-down therapy for patients who have responded well to amphotericin B. Elimination of predisposing factors for infection, such as hyperglycemia, metabolic acidosis, immunosuppressive drugs, and neutropenia, is also crucial. The overall mortality of mucormycosis in case series from India is 15-31% [14].

## Case presentation

Case one

A 70-year-old gentleman with uncontrolled diabetes mellitus presented with left eye swelling and proptosis, nasal discharge, and bilateral nasal obstruction. One month before, he was positive for COVID-19, which was managed with broad-spectrum antibiotics and intravenous corticosteroids for a duration of 12 days. A tissue specimen was obtained from the left nasal cavity and was suggestive of mucormycosis. Two weeks after the diagnosis of mucormycosis, the patient developed right-sided sudden onset weakness, left-sided seventh nerve palsy, and slurring of speech. Random blood sugar (RBS) at the time of admission was 225 mg/dl. Magnetic resonance imaging (MRI) of the brain, orbits, and paranasal sinuses revealed left-sided erosions of medial and inferior walls of the orbit with involvement of intra- and extraconal fat, bulky muscles displaying heterogenous post-contrast enhancement, optic neuritis, superior orbital fissure, orbital apex involvement, proptosis, and panophthalmitis. Left-sided cavernous sinus infiltration with loss of flow voids in C2, C3, and C4 in the internal carotid artery (thrombosis) and acute lacunar infarcts in cerebral hemispheres, gangliocapsular region, and corona radiata were present. Right and left-sided maxillary, ethmoid, sphenoid, frontal, and superior alveolar processes were involved. The patient was administered liposomal amphotericin B, posaconazole, and trans-cutaneous retrobulbar amphotericin-B (TRAM-B). Orbital exenteration and endonasal endoscopic debridement were performed. A few days after surgery, the patient developed sudden deterioration in consciousness with a Glasgow coma scale (GCS) score of 3/15 and was transferred to the infectious diseases intensive care unit (ICU), where he was intubated and ventilated. The patient died on the same day.

Case two

A 67-year-old gentleman with diabetes mellitus controlled on oral hypoglycemics presented with left eye swelling and restricted movements, headache, pain in the lower jaw, and loss of sensation on the left side of the face. One month earlier, the patient had developed fever and shortness of breath, for which he was administered oral steroids. A reverse transcriptase-polymerase chain reaction (RT-PCR) done on admission confirmed the COVID-19 positive status of the patient. On examination, left-sided lower motor neuron facial nerve palsy was present. On torchlight examination, periorbital edema and chemosis were present. A tissue specimen was obtained from the left nasal cavity, and mucormycosis was diagnosed. Biochemical investigations revealed increased values of C-reactive protein (CRP) (38.9 mg/L), procalcitonin (0.8 ng/ml), serum ferritin (433 ng/ml), fibrinogen (489 mg/dl), RBS (174 mg/dl), haemoglobin (Hb) (9.4 g/dl), and mild leucocytosis (13,000). MRI of the brain, orbits, and paranasal sinuses revealed left-sided nasolacrimal duct involvement, erosions of medial and inferior walls of the orbit with involvement of intra- and extraconal fat and extension up to preseptal compartment, bulky muscles displaying post-contrast enhancement, optic neuritis, superior orbital fissure, and orbital apex involvement. Left-sided cavernous sinus infiltration and an enhancing dural thickening along the anterior temporal lobe were present. Right and left maxillary, ethmoid, sphenoid, and frontal sinuses were involved with right-sided erosion of anterior and medial walls. The patient was administered liposomal amphotericin B, posaconazole, isauvaconazole, and TRAM B. Endonasal endoscopic debridement was performed. The patient was discharged after two months. The patient has maintained regular follow-ups since then and has been taking posaconazole.

Case three

A 42-year-old gentleman presented with a headache, pain in the left eye, and diplopia for the past 20 days. The patient had a history of treatment with broad-spectrum antimicrobials for an upper respiratory tract infection one month prior to the development of symptoms of mucormycosis. The patient was never tested for COVID-19 before presentation, where RT-PCR for COVID-19 was done and was negative. The patient was not a known case of diabetes and had no history of steroid administration. On torchlight examination of eye, periorbital edema and chemosis were present. A tissue specimen was obtained from the left nasal cavity, and mucormycosis was diagnosed. Biochemical investigations revealed increased values of CRP (20.5 mg/L), fibrinogen (425 mg/dl), decreased Hb (9 g/dl), and leucocytosis (14,500). The patient was administered antibacterials for sinusitis, liposomal amphotericin B, and posaconasole. Three injections of TRAM B were given, and endoscopic endonasal debridement was performed. MRI of the brain, orbits, and paranasal sinuses revealed left-sided erosion of medial and inferior walls of the orbit with involvement of intra- and extraconal fat and extension up to the preseptal compartment, bulky muscles displaying post-contrast enhancement, optic neuritis, superior orbital fissure, and orbital apex involvement. Cavernous sinus infiltration and an enhancing dural thickening along the anterior temporal lobe were present on both left and right sides. Right and left maxillary, ethmoid, sphenoid, and frontal sinuses were also involved. The patient was discharged after two months and is currently alive and well and is compliant with regular follow-up.

Case four

A 50-year-old gentleman with newly diagnosed diabetes presented to us with headaches, diminished vision in the right eye, and diplopia for the past 15 days. One and a half months before admission, he developed a fever and cough and subsequently tested positive for COVID-19 on RT-PCR. He was treated with supplemental oxygen through a nasal cannula and oral steroids for 10 days. Upon admission for the following symptoms, he tested negative on RT-PCR. On examination, right periorbital edema and right cheek swelling were noted. The nasal septum was deviated to the right, and discharge was present in the right nasal cavity. The right eye was nonreactive to light, and the pupil was dilated. Ocular movements in the right eye were restricted in all gazes. Nasal debridement was performed, and the tissue sample was sent for histopathology, which confirmed the diagnosis of mucormycosis. Lab investigations revealed elevated CRP (29 mg/L), d-dimer (0.6 ug/L), lactate dehydrogenase (LDH) (586 IU/L), ferritin (323 ug/L), IL-6 (26 pg/ml), and procalcitonin (0.2 ng/ml); Hb (11.1 g/dl) was reduced. MRI of the brain, orbits, and paranasal sinuses revealed right-sided optic neuritis, bulky ocular muscles displaying post-contrast enhancement, orbital apex involvement, and mucoperiosteal erosion of the medial wall of the left orbit with extraconal fat stranding. The right cavernous sinus was diffusely infiltrated, flow void was noted in the right ICA, dural enhancement was noted along the right base frontal lobe, and frontal and temporal lobe cerebritis was present. Right and left maxillary, ethmoid, sphenoid, and frontal sinuses were involved. The patient was administered antibacterials for sinusitis, liposomal amphotericin B, posaconazole, and hyperbaric oxygen therapy. Three injections of TRAM B were given, and endoscopic endonasal debridement was performed. The patient was discharged after two and half months and is currently alive and well. He is still taking posaconazole syrups and is compliant with follow-up.

Case five

**Figure 1:**
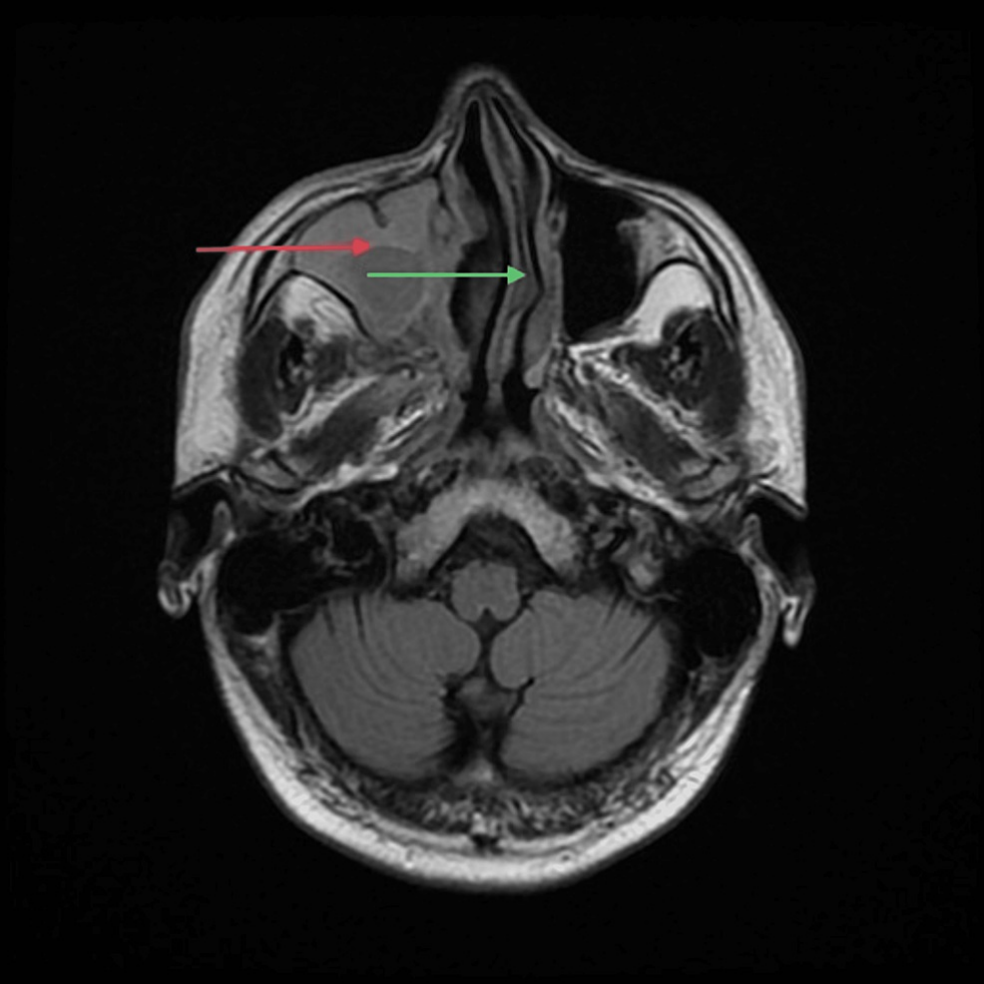
T1-weighted MRI head (axial section) of Case Five showing right maxillary sinusitis and infiltration (red arrow), and nasal septum deviation (green arrow)

**Figure 2:**
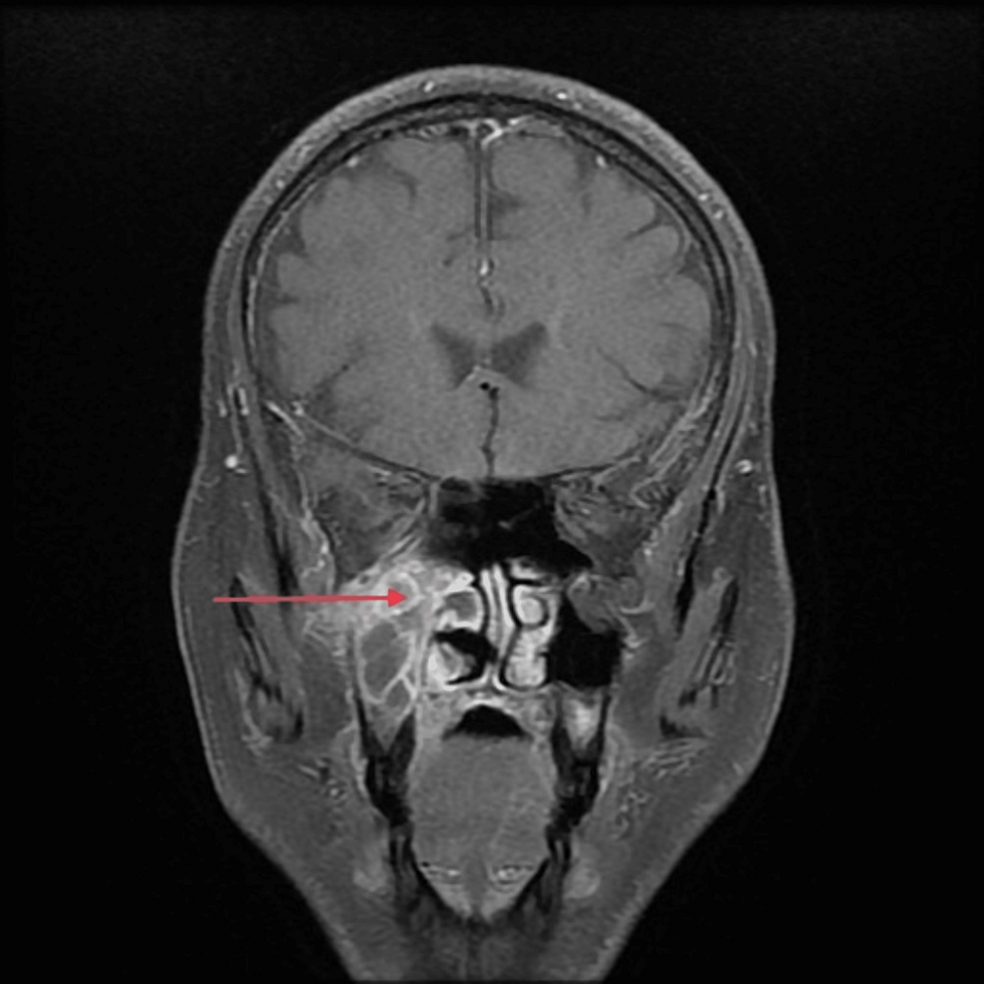
T1-weighted MRI head (coronal section) of Case Five showing right maxillary sinusitis and infiltration (red arrow)

A 44-year-old gentleman with a history of diabetes for the past 10 years (controlled on oral hypoglycemics) presented to us with bilateral loss of vision and pain in the upper jaw for 28 days. He had contracted COVID-19 a month prior to presentation and tested positive on RT-PCR. Subsequently, he developed COVID-19 pneumonia and was treated with broad-spectrum antimicrobials along with oral steroids for four days. Upon admission for the following symptoms, he tested negative on RT-PCR. On examination, a diffuse swelling was noted on the right side of his face, which was tender to touch. Periorbital edema was present. Nasal debridement was performed, and the tissue sample was sent for histopathology, which confirmed the diagnosis of mucormycosis. Lab investigations revealed random plasma glucose of 180 mg/dl, reduced Hb (11.3g/dl), CRP (48 mg/dl), and d-dimer (0.8 ug/ml). MRI of the brain, orbits, and paranasal sinuses revealed right-sided erosions of medial and inferior walls with involvement of intra- and extraconal fat, optic neuritis, superior orbital fissure, and orbital apex involvement. Focal infiltration of the cavernous sinus and cribriform plate involvement were present on the right side. Right-sided maxillary sinus was involved with soft tissue filling in the maxillary antrum, causing erosion of medial, lateral, and anterior walls. The ethmoid sinus and sphenoid sinus were involved with the involvement of the greater wing. The superior alveolar process is involved. Left-sided maxillary and sphenoid sinus were involved. The T1-weighted MRI head findings of this case are displayed in Figures 1, 2. The patient was administered antibacterials for sinusitis, liposomal amphotericin B, posaconazole, and TRAM B. Endoscopic endonasal debridement was performed. The patient was discharged after two and half months and is currently alive and well. He is still taking posaconazole syrups and is compliant with follow-up.

The clinical and demographic profiles along with the initial complaints and MRI findings of the five cases of rhino-orbito-cerebral mucormycosis are given in Tables 1, 2.

**Table 1:** Clinical and demographic profiles and risk factors of rhino-orbito-cerebral mucormycosis patients CNS: central nervous system; AKI: acute kidney injury; PNS: para-nasal sinuses; COVID-19: coronavirus disease 2019

Parameters	Case 1	Case 2	Case 3	Case 4	Case 5
Age	70	67	42	50	44
Sex	Male	Male	Male	Male	Male
History of COVID-19	Yes	Yes	No	Yes	Yes
History of broad-spectrum antimicrobials	Yes	Yes	Yes	Yes	Yes
History of diabetes mellitus	Yes	Yes	No	Yes	Yes
Obesity	No	No	No	Yes	No
History of hypertension	No	Yes	No	Yes	No
History and duration of oral steroids	No	Yes	No	Yes	Yes
History of IV steroids	Yes	No	No	No	No
Occular involvement	Yes	Yes	Yes	Yes	Yes
CNS involvement	Yes	Yes	Yes	Yes	Yes
AKI during hospital stay	No	Yes	No	No	Yes
Anaemia during hospital stay	No	Yes	No	No	Yes
PNS involvement	Yes	Yes	Yes	Yes	Yes
Outcome	Death	Survived	Survived	Survived	Survived

**Table 2:** Initial complaints and MRI findings of rhino-orbito-cerebral mucormycosis patients CNS: central nervous system; PNS: para-nasal sinuses; ICA: internal carotid artery

Cases	Initial complaints	Occular involvement	CNS involvement	PNS involvement
CASE 1	Left eye swelling, bilateral nasal obstruction, and nasal discharge	Left-sided: Erosion of medial and inferior walls and involvement of intra- and extraconal fat. All muscles are bulky and are displaying post-contrast enhancement. Optic neuritis present, and superior orbital fissure and orbital apex involved. Panophthalmitits present.	Left-sided: Cavernous sinus infiltration and loss of flow voids in C2, C3, and C4 in ICA; thrombosis present. Acute lacunar infarcts in cerebral hemispheres, gangliocapsular region and corona radiata present.	Right and left-sided maxillary, ethmoid, sphenoid and frontal sinuses and superior alveolar process involved.
CASE 2	Left eye swelling, restriction of movement of left eye, and deviation of angle of mouth to the right side.	Left-sided: Nasolacrimal duct involved. Erosions of medial and inferior walls with involvement of intra- and extraconal fat and extension up to preseptal compartment. All muscles bulky, displaying post-contrast enhancement. Optic neuritis present and superior orbital fissure and orbital apex involved.	Left-sided: Cavernous sinus infiltration and enhancing dural thickening along anterior temporal lobe present.	Right and left-sided maxillary, ethmoid, sphenoid, frontal sinuses involved.
CASE 3	Headache, left-sided uniocular diplopia, and pain around left eye	Left-sided: Erosion of medial and inferior walls and involvement of intra- and extraconal fat. All muscles are bulky and are displaying post-contrast enhancement and extension up to preseptal compartment. Optic neuritis present and superior orbital fissure and orbital apex involved.	Left-sided: Cavernous sinus infiltration and enhancing dural thickening along para cavernous region present. Cribriform plate eroded. Right-sided: Enhancing dural thickening along para cavernous region. Cribriform plate focally eroded.	Right-sided: Maxillary, ethmoid and sphenoid sinuses involved. Left-sided: Maxillary, sphenoid, ethmoid and frontal sinuses involved.
CASE 4	Right sided headache, and right sided loss of vision	Right-sided: All ocular muscles bulky, displaying post-contrast enhancement. Optic neuritis present and orbital apex involved. Left-sided: Mucoperiosteal erosion of medial wall of left orbit with extraconal fat stranding..	Right-sided: Diffusely infiltrated right cavernous sinus. Flow void noted in Right ICA. Dural enhancement noted along right basifrontal lobe. Frontal and temporal lobe cerebritis present.	Right and left maxillary, ethmoid, sphenoid, frontal sinuses involved.
CASE 5	Blurring of vision right eye, and pain in upper right jaw.	Right-sided: Erosions of medial and inferior walls with involvement of intra- and extraconal fat. Optic neuritis present and superior orbital fissure and orbital apex involved.	Right-sided: Focal infiltration of cavernous sinus and cribriform plate involved.	Right-sided: Maxillary sinus involved with soft tissue filling in maxillary antrum causing erosion of medial, lateral and anterior walls. Ethmoid sinus involved. Sphenoid sinus involved with involvement of greater wing superior alveolar process. Left-sided: Maxillary sinus and sphenoid sinus involved.

## Discussion

The second wave of COVID-19 in India did not only present with acute infections and post-infective pneumonia but also opportunistic infections in patients who were either immunocompromised, uncontrolled diabetics, or treated with high-dose oral or intravenous steroids. The incidence rate of mucormycosis globally varies from 0.005 to 1.7 per million population [15]. In India, the prevalence of mucormycosis is estimated at 140 per million population, which is about 80 times higher than the prevalence in developed countries [16]. In comparison with the background prevalence of mucormycosis in comorbid populations, the prevalence of COVID-19-associated mucormycosis was 50 times higher than the highest documented prevalence of mucormycosis that was among diabetic patients in India (0.14 cases per 1000 patients) [16].

Prior to the COVID-19 pandemic, mucormycosis was most commonly reported in severely immunocompromised patients with uncontrolled diabetes [1]. In some patients, it was a diabetes-defining illness. The most common clinical presentation of mucormycosis is rhino-orbital-cerebral infections. Hyperglycemia, usually with an associated metabolic acidosis, is the most common underlying condition [1]. The infection usually presents as acute sinusitis with fever, nasal congestion, purulent nasal discharge, headache, and sinus pain. All of the sinuses become involved and spread to contiguous structures, such as the palate, orbit, and brain usually progress rapidly over the course of a few days. The hallmarks of spread beyond the sinuses are tissue necrosis of the palate resulting in palatal eschars, destruction of the turbinates, perinasal swelling, erythema, and cyanosis of the facial skin overlying the involved sinuses and/or orbit. Signs of orbital involvement include periorbital edema, proptosis, and blindness. Facial numbness is frequent and results from infarction of sensory branches of the fifth cranial nerve. Spread from the sphenoid sinuses to the adjacent cavernous sinus can result in cranial nerve palsies, thrombosis of the sinus, and involvement of the carotid artery. Diagnosis is made by obtaining specimens during endoscopic evaluation, which are inspected for broad, non-septate hyphae with right-angle branching. Further evaluation for the extent of spread of the infection is done by computed tomography (CT) or MRI. Several cases were admitted to the mucormycosis wards in our institute in April-July 2021 and were considered a breakthrough in the incidence of this rare type of fungal infection.

In this case series, we reported five cases of post-COVID 19 mucormycosis that presented with sinusitis and focal neurological deficits to the medicine department of King George’s Medical University during the second wave of COVID-19 pandemic in India in the months of April-May, 2021. All five were above 40 years of age and had mucormycosis confirmed by histopathology. Four out of five patients had a history of or were diagnosed with COVID-19 infection when they presented with symptoms of mucormycosis. All of them had a history of administration of broad-spectrum antimicrobials. All the cases had a history of non-invasive oxygen supplementation. Of the patients, 80% were previously diagnosed as diabetics and/or were treated with high-dose corticosteroids for COVID 19; both are considered risk factors for immunosuppression. Insulin was used to treat the deranged glycemic status caused by the use of high-dose steroids. Two of these cases were known cases of hypertension, and one of them had obesity. All the cases had disease involvement in the eye, CNS, and paranasal sinuses. Reduction in visual acuity in cases with mucormycosis is explainable on the basis of infarctions in blood vessels supplying the retina or optic nerve, compression on the nerve along its course within the cavernous sinus, or direct infection and necrosis [12,13]. Optic neuritis, orbital apex involvement, and cavernous sinus infiltration were present in all the cases. Patient one, who developed right hemiparesis and left upper motor neuron (UMN) seventh nerve palsy, also had acute lacunar infarcts in cerebral hemispheres, gangliocapsular region, and corona radiata. All the patients were treated with multiple doses of antimicrobials, liposomal amphotericin B, posaconazole/isavuconazole syrups, and TRAM B. Hyperbaric oxygen therapy was given to patient two and patient four. Endoscopic nasal debridement was performed on all patients. Orbital exenteration was only performed on Patient one. Patients two and five were operated on by the oro-maxillo-facial surgery (OMFS) department. Case series from India exhibit overall mortality of 15-31% in cases of mucormycosis [14]. In this case series, the mortality was 20%.

According to the World Health Organisation (WHO), COVID-19 vaccines provide strong protection against serious illness, hospitalization, and, therefore, the development of mucormycosis later [17].

## Conclusions

Mucormycosis is a very rare phenomenon, but during the second wave of the COVID-19 pandemic in India, it was unfortunately widespread and associated with significant morbidity and mortality. The major learning point is that in a patient with recent history of COVID-19 infection, uncontrolled diabetes mellitus, and high-dose steroid supplementation are the most important risk factors for the development of mucormycosis.

The essential elements for successfully managing this fatal infection are controlling the predisposing factors, early detection with a high index of suspicion in patients with contributing factors, anti-fungal drugs, and surgical debridement of the involved tissues. Amphotericin-B-associated nephrotoxicity is a severe cause of morbidity during the treatment of mucormycosis patients. Hence, such patients should receive special care by adequate hydration and regular renal function tests. COVID-19 vaccines provide strong protection against serious illness, hospitalization, and, therefore, the development of mucormycosis later.
